# The malignant niche: safe spaces for toxic stem cell marketing

**DOI:** 10.1038/s41536-017-0036-x

**Published:** 2017-12-19

**Authors:** Douglas Sipp

**Affiliations:** 1 RIKEN Center for Developmental Biology, Kobe, Japan; 2 Keio University School of Medicine, Tokyo, Japan; 3 Keio University Global Research Institute, Tokyo, Japan; 4 RIKEN Center for Advanced Intelligence Project, Tokyo, Japan

## Abstract

Many tumors are sustained by microenvironments, or niches, that support and protect malignant cells, thus conferring a competitive advantage against both healthy cells and therapeutic interventions (for a brief review, see Yao and Link (Stem Cells 35: 3–8, 2017)). The global industry engaged in the commercial promotion of unproven and scientifically implausible cell-based “regenerative” therapies has developed a number of self-protective strategies that support its survival and growth in ways that are broadly analogous to the functions of the malignant niche.

## Background

The first media reports of the marketing of stem cells for^1^ unsupported therapeutic use emerged in the early 2000s.^2,3^ The promotional practices of these early companies, many of which were based in the United States and Western Europe, tapped into the public fascination with the apparently revolutionary medical potential of cell-based strategies, but also a growing sense of frustration that government regulation was impeding innovation and preventing rapid access to breakthrough therapies by patients with serious unmet medical needs. In the US, this frustration was compounded by the 2001 Bush administration restriction of federal funding of new human embryonic stem cell lines created after August 2001, which was widely misunderstood as a “ban” on stem cell research. In the face of several of widely publicized closures of stem cell businesses and prosecution of the owners by law enforcement authorities, such as the federal law enforcement actions against the proprietors of Biomark (later Advanced Cell Therapeutics)^4^ and Stowe BioTherapy Inc.,^5^ a second wave of stem cell marketing operations emerged. These businesses, which were often based in developing economies, targeted both local residents and patients from wealthier nations, giving rise to what became known for a time as “stem cell tourism”.^6,7^ Such businesses offered cell-based or tissue-based procedures, sometimes targeting more than 100 different medical conditions, at prices of tens of thousands of dollars per treatment course. Due to vague or non-existent regulation, or lax enforcement, numerous such companies remain in operation in countries, such as Mexico, India, and Thailand.^8^

More recently, however, businesses marketing unproven cell interventions have re-emerged in the developed world, building thriving “regenerative” therapeutics marketing industries in nations, which are typically considered at the forefront of biomedical innovation and regulation.^9–11^ In this brief review, I will examine some of the strategies used by such businesses, individually and collectively, to support their own growth and insulate themselves against regulatory oversight. In the interests of space, my focus will be on firms promoting unorthodox uses of self-described “stem cells”. I note, however, that similar observations could be made for other industries marketing unproven regenerative biologics, such as certain forms of cancer “immunotherapy,” anti-aging lotions and supplements, fetal tissue transplants, so-called “fresh cell” injections of lyophilized fetal animal tissue, or the use of platelet-rich plasma in orthopedic and other conditions. An understanding of how these protective niches are established and maintained will be essential to developing effective countermeasures against malignant industry behaviors.

## Regulatory evasion

The 2016 report by Turner and Knoepfler on the state of stem cell marketing in the United States^10^ shed light on a phenomenon that caught many in the regenerative medicine field by surprise. The revelation that more than 350 business were selling unproven stem cell interventions at 570 clinical sites caused many to question how this could occur in a country with the world’s largest biomedical regulatory agency, the Food and Drug Administration (FDA). One key to the industry’s growth has been the exploitation of a once obscure provision in the federal code, CFR 1271.15(b), which states:

You are not required to comply with the requirements of this part if you are an establishment that removes HCT/P’s [Note: This abbreviation refers to “human cell and tissue products,” a defined subset of which are regulated as biological drugs by FDA.] from an individual and implants such HCT/P’s into the same individual during the same surgical procedure

To my knowledge, the first business to explicitly refer to this exception in its promotional materials is Regenerative Sciences, a Colorado firm that engaged in lengthy but ultimately unsuccessful litigation against FDA over its marketing of a cultured bone marrow-derived cell product. During the course of this litigation, the company introduced a “same-day” version of the cultured product, which it continues to offer in its Colorado office. The FDA won a permanent injunction against the marketing of the cultured cell product in 2012, and the company offshored this service to a subsidiary located in the Cayman Islands. Since that time, the “same surgical procedure exception” has become the first line of defense against regulatory oversight by companies in the US.

Other economically developed nations with thriving stem cell marketing industries, such as Japan and Australia, similarly broad latitude is given to the use of certain forms of cellular biologics in private practice by medical professionals. In Australia, the Therapeutic Goods Administration currently does not exert oversight over autologous cell-based interventions of any sort, whereas in Japan private medical practitioners are empowered to use certain “regenerative medicines” after only nominal institutional review.^12^ The EU has recommendations for regulatory exceptions for “named patient” and “hospital exemption” programs for advanced therapy medicinal products (ATMPs).^13^ The former allows national authorities to approve access to investigational products for individual patients who have exhausted treatment options but do not qualify for inclusion in a clinical trial. The latter sets rules for applications of ATMPs “prepared on a non-routine basis according to specific quality standards, and used within the same Member State in a hospital under the exclusive professional responsibility of a medical practitioner, in order to comply with an individual medical prescription for a custom-made product for an individual patient.” While application of these rules varies considerably in member nations, enforcement actions against clinics in UK, the Netherlands, Germany, and Italy have helped to temper the growth of the illicit market.

In 2014, FDA introduced a draft guidance document in which it set out to clarify the definition of the same surgical procedure exception, limiting it processes in which the cell or tissue product is cleansed, rinsed, sized, or shaped ex vivo. However, this document remains in draft and its prospects for implementation under a government administration that has committed to reducing regulation across the board remain murky. In August 2017, the FDA Commissioner issued a statement of intent^14^ to clarify the agency’s policies on regenerative medicine, referring specifically to the problem of exploitation of patients by “dishonest actors,” and the need to bring the FDA into line with the recently enacted 21st Century Cures Act, which introduced both a new accelerated approval pathway for regenerative medicine products and called for the expanded use of “real world evidence” in agency pre-market reviews of medical products. The latter provision has attracted some criticism due to concerns that real world evidence may be of inferior quality to that derived from randomized clinical trials.^15^ However, the details of future FDA policy in this space remain indeterminate at the time of writing.

The challenges facing regulatory efforts by the federal government are compounded by the increasingly activist role of state governments in passing legislation that seeks to “nullify in practice” federal laws. The torrent of so-called “Right to Try” laws successfully promoted by the Goldwater Institute, a free market-oriented think tank, in at least 34 states is among the most prominent examples affecting investigative medical products in general. However, a number of states have also sought to undercut federal authority with specific respect to experimental regenerative medicine interventions. Texas is the most notable case of state-level dysregulation.^16^ In 2012, the Texas Medical Board approved a motion to allow state-based providers to offer investigational cell-based interventions with only an approval from an institutional review board, seemingly in a bid to bypass FDA oversight. In 2017, the Texas state legislature further passed a bill to allow physicians to deliver investigational “adult stem cell” interventions to patients with serious chronic disease or terminal illness without FDA authorization.^17^ The role of stem cell marketing businesses and affiliated groups in lobbying for such deregulation has been visible in the coordinated opposition to FDA authority in this space witnessed at public hearings and online comment forums in 2016.^18^

## Hijacking signaling pathways

A second component to the growth of the US industry has been its ability to use both mainstream and social media to promote its ends. Such practices clearly leverage and build on a playbook of persuasive and self-indemnifying strategies that has evolved in the marketing of other unorthodox medicines.^19^ Independent of, but complementary to, the media manipulation undertaken by private businesses, the field of stem cell research has a well-documented “hype” problem, in which early research findings are too often trumpeted as breakthroughs with profound therapeutic implications by media outlets, public communications officers, and scientists themselves.^20^ Building on this foundation of inflated expectation, stem cell providers make extensive use of human interest reports in local newspapers, and generalist magazines, proclaiming their miraculous successes or providing a forum for families to make fundraising pleas for expensive procedures.^21^ As in other industries, stem cell business’s online media presence is central to their marketing portfolio, taking the form of attractive websites with seductively phrased promotional messages. Many firms also sponsor blogs and social media spaces where groups of followers, including past and prospective patients interact with employees.

The effects of these concerted engagement efforts have been key to fostering a public image of stem cell marketers as beneficent mavericks who side with desperately ill patients against misguided government interference in the “search for a cure.” This online media barrage approach was pioneered by early firms, such as TheraVitae in Thailand and Beike Biotechnology in China, which listed or linked to numerous but otherwise undocumented testimonials of successful stem cell cures; both firms employed American marketing representatives to lead their marketing campaigns.

During a 2016 public hearing concerning four draft guidance documents under consideration by FDA (including the one clarifying the same surgical procedure exception described above), representatives of stem cell marketing companies were joined by a chorus of patients decrying the proposed changes, and the general principle that human cell and tissue products should be subject to any federal oversight whatsoever. An online comment forum also established by FDA was flooded with thousands of comments in opposition to federal regulation. However, the large number of identical or near-identical submissions is suggestive of a coordinated effort to bias the outcome of the call for comments.^18^

The success of the industry’s communications strategies is attested to by the strong sense of identification many individuals have voiced with stem cell businesses, frequently expressed as an “us vs. them” fight against an incompetent or corrupt government that has been captured by the interests of the biomedical research establishment and/or “Big Pharma.”

## Co-optation of resources

In addition to gaming the legal system and manipulating public opinion, the stem cell marketing industry has also profited enormously from its use of resources developed with public funds. The most common instance of this is the citation of academic research, much of it at basic or preclinical stages, to signal that stem cell-based interventions are on the verge of entering mainstream medical practice, often with the additional suggestion that the sole remaining roadblock is intrusive regulation by the state. Numerous industry-funded websites or online newsletters aggregate links to academic stem cell research articles with no apparent connection to the site’s corporate sponsor. A number of firms have additionally self-published books with extensive bibliographies referencing publicly funded studies as evidence of therapeutic efficacy; one such book by a (now defunct) Korean firm with subsidiaries or affiliates in California and Texas, as well as Japan, China, and Germany, published a promotional book with nearly 150 pages of references, nearly all of which cite government-funded basic research studies.

## Metastatic potential

In cancer, the malignant niche is a localized phenomenon and the stem cell marketing industry activities outlined above exhibit similar self-protective effects for individual businesses or the industry as a whole. While individual companies, industry organizations, and industry-funded “astroturfing” campaigns have all contributed to the promulgation of messages seeking to align customers with the business interests that profit from their exploitation, the role of private policy groups dedicated to opposing government intervention in markets in any form cannot be neglected.^22^ Such organizations have historically played central roles in promoting resistance to regulation of tobacco products and climate change countermeasures,^23^ as well as to general opposition of FDA authority in regulating drug efficacy studies.^24^

There is a body of evidence that such policy groups, in cooperation with like-minded political groups, supported stem cell deregulation as a wedge issue in their efforts to weaken regulatory authority over medical products in general.^22,25^ Individuals associated with the Manhattan Institute, a free market think tank, which has long called for weakening of the FDA, for example, published editorials in business publications and issued a 36-page legal policy report during case brought by Regenerative Sciences against the FDA, described above. In Japan, the introduction of the conditional approval pathway for regenerative medicine products mirrored in part proposals made in “Free to Choose Medicine,” a book published by a Heritage Foundation scholar, which advocated for allowing medical products of any kind to be purchased after Phase I trials. In 2017, the Japanese Ministry of Health, Labour, and Welfare announced plans to expand conditional approvals to the entire range of product categories regulated by the Pharmaceuticals and Medical Devices Agency.^26^

The influence of privately free-market and libertarian think tanks in lobbying for deregulation of regenerative medicine should remain an area of interest and concern both for the field and for the wider global health community due to the potential of metastasis of a currently local malignant phenomenon.

## Developing interventions

As alluded to in the title, the behavior of stem cell businesses that lack credible evidence to support their therapeutic claims and actively resist efforts intended to test their products for efficacy in some ways resemble the self-protective behaviors of malignant cells, which are notoriously well-defended against immune surveillance, and resistant to remedy. The War on Cancer was launched in the early 1970s and, despite notable successes, continues today, a testament to the durability of pathologic distortions of the body’s own systems. The distorted marketing of nominal “regenerative medicine” interventions has shown a similar robustness and sophistication in evading regulation and enforcement. While no single “cure” may be possible, scientists, physicians, journalists, and regulators should arm themselves with a knowledge of how these businesses promote their own growth and survival at the expense of the body politic.

Active and well-coordinated responses by a community of stakeholders will be needed to counter the growth of this dysfunctional market. Professional organizations, such as the International Society for Stem Cell Research and the International Society for Cell Therapy have issued public warnings and practice guidelines, which provide a much-needed framework of reference standards and ethical norms to guide the field. A number of medical organizations have also issued alerts on the potential for exploitation and harm by unscrupulous stem cell businesses, and counseled their members on how to advise patients who may be considering an unproven regenerative treatment.^27^ Researchers have cautioned against the enabling effects of hype and exaggerated claims of near-term therapeutic potential when reporting early research results.^20^

But such self-regulatory efforts alone are unlikely to stanch the proliferation of the lucrative stem cell marketing industry. The FDA’s announcement of plans to crack down on dubious clinics is a welcome signal. Actions by state and federal law enforcement agencies have also proven effective in several past cases. Medical licensing boards may also be effective through disciplinary actions, such as issuing warnings or revoking licenses to practice. Civil litigation, such as class action or individual lawsuits may also be effective in cases of fraud or malpractice. Internationally, the response to the stem cell dark economy might best be coordinated by global agencies, such as the World Health Organisation.^28^

As outlined above, businesses involved in the promotion of unproven and implausible stem cell “cures” have been remarkably effective at protecting their interests at the expense of the field, health care systems, and most importantly, individual patients. Effective countermeasures will be crucial to ensure that this unhealthy enterprise is contained and, ultimately, eradicated.
